# Self-Organizing Distributed Architecture Supporting Dynamic Space Expanding and Reducing in Indoor LBS Environment

**DOI:** 10.3390/s150612156

**Published:** 2015-05-25

**Authors:** Seol Young Jeong, Hyeong Gon Jo, Soon Ju Kang

**Affiliations:** School of Electronics Engineering, College of IT Engineering, Kyungpook National University, 80 Daehakro, Bukgu, Daegu 702-701, Korea; E-Mails: snowflower@ee.knu.ac.kr (S.Y.J.); tsana@ee.knu.ac.kr (H.G.J.)

**Keywords:** self-organizing dynamic space, indoor location-based service, distributed system architecture

## Abstract

Indoor location-based services (iLBS) are extremely dynamic and changeable, and include numerous resources and mobile devices. In particular, the network infrastructure requires support for high scalability in the indoor environment, and various resource lookups are requested concurrently and frequently from several locations based on the dynamic network environment. A traditional map-based centralized approach for iLBSs has several disadvantages: it requires global knowledge to maintain a complete geographic indoor map; the central server is a single point of failure; it can also cause low scalability and traffic congestion; and it is hard to adapt to a change of service area in real time. This paper proposes a self-organizing and fully distributed platform for iLBSs. The proposed self-organizing distributed platform provides a dynamic reconfiguration of locality accuracy and service coverage by expanding and contracting dynamically. In order to verify the suggested platform, scalability performance according to the number of inserted or deleted nodes composing the dynamic infrastructure was evaluated through a simulation similar to the real environment.

## 1. Introduction

An indoor location-based service (iLBS) [1] is becoming more prevalent owing to increase in the number of smartphone users. The infrastructure for iLBS systems must support dynamically inserted or deleted nodes, as well as reconfiguration for supporting an iLBS in the changed environment because the nodes in the iLBS system is used as a reference position. The building of infrastructure system in the indoor environment is not easy, and the required positioning accuracy depending on the service is very different. The degree of precision of localization in an iLBS depends on the number of anchor nodes in the cell-based location system [2]. Therefore, an infrastructure configured with a number of anchor nodes is different depending on the service domain, and is dynamic according to the environmental situation. In addition, each anchor node may need to be installed or removed anywhere and anytime regardless of the current infrastructure. Deleting an existing node can occur at any time, under both normal and abnormal situations. 

Global knowledge in a traditional centralized server is an obstructive factor in a dynamic iLBS system. However, many location systems have a location server for positioning mobile resources, which analyzes and calculates coordinates from the collected signal strength in a database [3,4,5]. A centralized architecture needs to maintain a complete geographic map of the entire building or complex in its central server, which can cause low scalability and traffic congestion. In order to solve these inherent challenges in the centralized server architecture, a location-based self-organizing fully distributed network infrastructure similar to a natural spontaneous environment with a lot of movement, such as an ant or bee colony, needs to be designed. Self-organization is a spontaneous process in which some form of global order or coordination arises out of the local interactions between the components of an initially disordered system. A self-organizing and fully distributed iLBS system would not need to maintain or employ a centralized map and would be dynamically adaptive to changes in the indoor infrastructure. In particular, the dynamic space must be reconfigured without influencing the overall infrastructure under transient circumstances. This is one of the self-organizational properties of the proposed architecture, which can support scalability of the physical space or of logical service groups.

The self-organizing service platform (SoSp) [6] was developed from our previous research for a distributed iLBS system. This paper proposes the more advanced self-organizing distributed architecture supporting a dynamic space that expands and contracts with a self-organizing approach based on the SoSp in a dynamic indoor environment. The Self-organizing Localized IoT Messaging (SLiM) Hub, which works as a unit of the overlay network [7] based on physical distance on top of the legacy physical network, is installed in a unit space such as a room or corridor in a building required for flexible space configuration or service such as a convention center. The SLiM Hub is used as a reference position of all devices and people in its unit space, and also possesses information on neighboring SLiM Hubs that are connected through a physical path such as a door or a corridor. The SLiM Hub also serves as a messaging hub for forwarding and push/pull IoT message among the mobile or resource devices.

The proposed fully distributed iLBS architecture improves the scalability of the infrastructure because there is no need to maintain a map of the entire building or complex in the central server. Dynamic reconfiguration functionality in this architecture can support scalability of the physical space or of logical service groups depending on various indoor location-based services. This paper constitutes the SoSp overlay network infrastructure, and discusses a method of configuring the dynamic space based on the proposed SoSp. 

The remainder of this paper is structured as follows. The approach towards an iLBS platform and basic concepts are discussed in Section 2, and Section 3 presents related works. Section 4 describes the proposed self-organizing distributed platform. Section 5 presents detailed operation principles of the dynamic space expansion and reduction in the proposed self-organizing distributed iLBS platform. Section 6 introduces implementation of the mobile devices and the SLiM Hub in the proposed platform, and demonstrates performance evaluation through a simulation. Finally, conclusions are drawn in Section 7.

## 2. Service Scenario and Proposed Approach

### 2.1. Indoor Location-Based Service Scenarios Using Proposed Approach 

**Figure 1:**
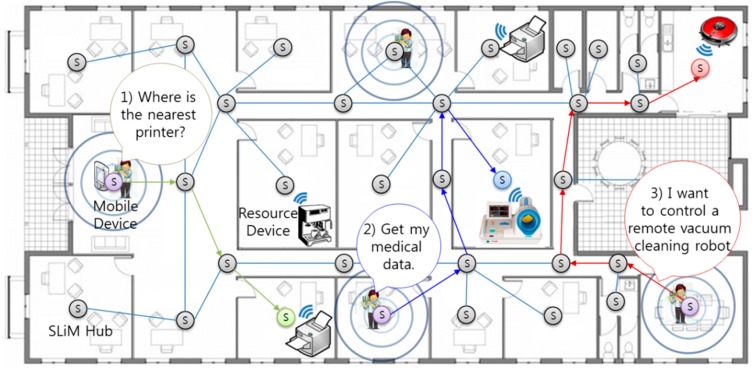
An example of an indoor location-based system for an office environment.

Figure 1 introduces examples of an indoor environment and opportunistic service scenario. There are numerous resource devices that are either mobile or stationary, with mobile devices like smartphones being able to request the following service examples:

(1) Lookup only scenario: A mobile device user can print to the nearest printer (resource device) from his current position without presetting (zero-configuration).
(2) Asynchronous request-reply scenario: After using a local sphygmomanometer, the person can look up and check his or her own medical data, such as blood pressure, anywhere and anytime regardless of current location without needing to configure any settings (delay-tolerance).
(3) Connection-oriented request-reply scenario: A user can remotely control a vacuum cleaning robot in real time, regardless of current location.

In order to provide these dynamic services among numerous mobile devices (e.g., a watch, smartphone, or smart pad) and service resource devices (e.g., a coffee machine, printer, sphygmomanometer, vacuum cleaning robot, TV, or file storage disk) in an indoor environment, an opportunistic iLBS network system (which works as an overlay network built on top of the legacy network) and an architecture for service binding are required. The cell-based SLiM Hub is attached (for example) to the ceiling in a room or a corridor and represents the position located within its range, as shown in Figure 1.

In addition, the SLiM Hub manages the connection between a service resource (e.g., the coffee machine) and a mobile device, such as a smartphone, and can also transfer the user’s private data, such as personal medical data, to particular SLiM Hubs or devices. The primary purpose of this research is to provide a dynamic iLBS between resource devices and mobile devices in this overlay network. In particular, numerous mobile devices can concurrently request (from several locations) services or resources that have dynamic and frequent mobility. Each SLiM Hub represents the position used to comply with requests for services located within its range. In this manner, the overall network architecture is fully decentralized and localized. In addition, expansion of the service area or an increase in the number of mobile or resource devices does not increase the complexity, unlike a traditional centralized server-based architecture.

In the proposed environment, the SLiM Hub has three roles: (1) anchor node for positioning in an iLBS system; (2) access point (AP) for various wireless communications, such as WiFi, Bluetooth (classic and low energy), ZigBee, and so on; (3) node for a connection point in an overlay network. First, the SLiM Hub represents the unit space of an installed location. Resource and mobile devices within this range can communicate with the SLiM Hub through diverse wireless communications. Finally, each SLiM Hub knows the distance to its directly connected neighbors, and can connect with a neighboring SLiM Hub.

### 2.2. Dynamic Space Expansion and Reduction 

**Figure 2:**
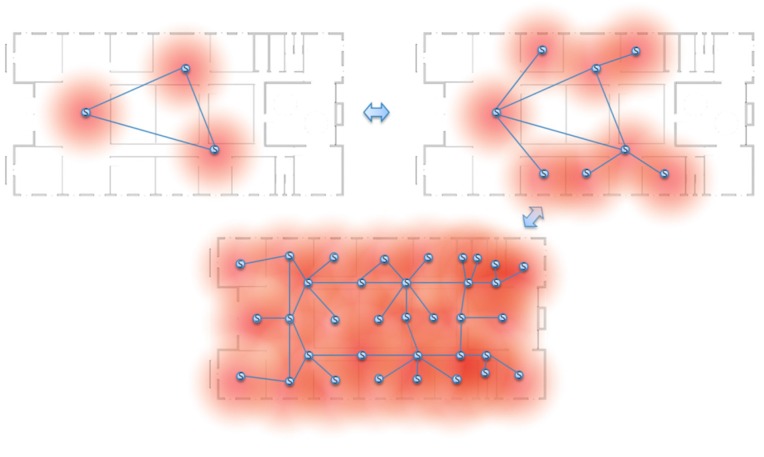
Dynamic space expansion and reduction.

The infrastructure of an iLBS system must be reconfigured when environment or type of supporting services change. The degree of precision of localization and the service coverage in an iLBS space depends on the number of anchor nodes, such as SLiM Hubs. If the system includes a small number of anchor nodes, the necessary message may be delivered, but it is difficult to provide accurate location-based services. If it needs a more precise location-based service and wants to expand the service area, the system requires the installation of more anchor nodes. Therefore, an infrastructure configured with a number of SLiM Hubs is different, depending on service domain and dynamic according to the environmental situation, as shown in Figure 2. In addition, each SLiM Hub may need to be installed or removed anywhere and anytime regardless of the current infrastructure.

The deletion of an existing SLiM Hub can occur at any time, in both normal and abnormal situations, and the dynamic space must be reconfigured without influencing the overall infrastructure under transient circumstances. This is one of the self-organizational properties of the proposed architecture, which can support scalability of the physical space or of logical service groups.

### 2.3. Overlay Network-Based Application Environment 

In order to provide natural networked services among numerous monitoring devices (e.g., a smartphone, smart pad, and so on) and resource devices (e.g., a person with a watch, wheelchairs, beds, and so on) in an iLBS environment, an iLBS network system, which works as an overlay network built on top of the legacy network, and an architecture for service binding are required. The cell-based SLiM Hub is expressed as a node in an overlay network graph. An edge in this graph signifies a physical path, such as a door or a corridor.

**Figure 3:**
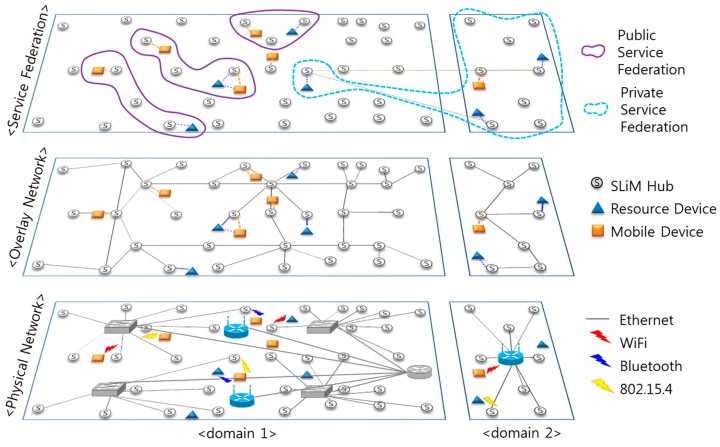
Service federations and an overlay network built on top of a physical network.

Figure 3 shows service federations and an overlay network built on top of a physical network. There are diverse service resources, such as health equipment, office equipment, or home appliances, in unit spaces such as a room, as shown in Figure 1. These service resources connect with the SLiM Hub, which is installed in this space, or mobile devices owned by users through wireless communications, such as WiFi, 802.15.4, BLE (Bluetooth Low Energy), ANT+, *etc.* Each SLiM Hub in this space has a list of neighboring SLiM Hubs interconnected through wired communication such as Ethernet. Connection with a neighboring SLiM Hub is described in Section 4.

Service federations are grouped by service range or user living space: public or private service federations. A public service federation is configured with geographically physical space, such as several related laboratory rooms in a building. On the other hand, a private service federation is configured by a personal user. For instance, smartphone users can set the specific service group for their offices and houses according to need. The broadcast communication manner of mobile agents in wireless sensor networks is not appropriate for a service federation, including physical or logical network connections on top of an overlay network. In addition, multitudinous mobile device users who feel a need for an iLBS can concurrently obtain opportunistic service resources from their respective locations. Consequently, a fully distributed infrastructure accommodates these numerous mobile devices in several positions as opposed to an approach based on centralized server architecture. Unnecessary traffic concentration in the centralized method can result in traffic congestion.

## 3. Related Works 

iLBSs need to increase their accuracy and know the precise localization of users or mobile devices, as well as determine an efficient infrastructure topology in an intelligent building [8]. In order to efficiently provide a dynamic iLBS, a well-designed infrastructure architecture platform is needed. 

As a well-known iLBS system, Apple Inc. is supporting an indoor positioning system called iBeacon [1] that works on BLE. In a real life scenario, it would be more of a location-aware, context-aware, pervasive small wireless sensor beacon that could pinpoint a user’s location in an indoor environment like a store. The iBeacon works by using BLE proximity sensing to transmit a universally unique identifier (UUID) picked up by a compatible app or OS that can be turned into a physical location or trigger an action on the device. A smartphone has plenty of battery capacity and scans the beacon signal to notice its current location in the iBeacon service. However, lots of mobile or wearable devices have only limited amount of battery power and need to last a long time after just one charge.

The other interesting LBS system [9] suggests a positioning technique based on interworking 3G and wireless network in heterogeneous environments such as indoor and outdoor. This LBS system can seamlessly provide a service tailored for each need: most convenient positioning technology in terms of precision cost trade-off; user-bearer communication technology available at a given moment in the considered area; and inter-WISP roaming capability. 

An iLBS system, in conjunction with the Cisco Mobility Services Engine (MSE) [3], is also a well-known commercial location system. MSE estimates the number of visitors, the amount of time they spend, and the frequency of their visits within the site. Additionally, it provides information about movement patterns by these visitors while they are in the building. However, in the MSE system based on a centralized architecture, a location server provides general location services for a network, and is primarily responsible for running the algorithms that predict client location. Therefore, the location server is also a single point of failure in the MSE system. Specifically, this paper shows the reconfiguring of the dynamic space in the distributed platform unlike this centralized server-based architecture.

On the other hand, a distributed architecture approach has also been suggested in related research. MobiEyes [10] presents a distributed architecture and a suite of optimization techniques for scalable processing of continuously moving location queries. The main idea behind the MobiEyes distributed architecture is to promote careful partition of a real-time location monitoring task in optimal coordination of server and client-side processing. In the MobiEyes system, the geographic area of interest is covered by several base stations, which are connected to the service provider’s server. Then, all location service requests are served through a three-tier architecture that consists of mobile objects, base stations, and the server. However, the MobiEyes system is not a fully distributed architecture, and even if the base stations act the part of multi-agents, a method of providing services based on a server system brings a traffic congestion problem and a single point of failure in a dynamic environment with numerous mobile objects.

In addition, some decentralized model studies include a self-organized and emerging overlay network and have been widely utilized to accomplish efficient resource discovery in various fields. One self-organizing traffic light system [11] possesses global synchronization achieved adaptively through local interactions between cars and traffic lights and generates flexible green-light waves on demand, unlike centralized legacy traffic lights systems. Apart from a self-organizing distributed concept in these various domains, there is research into configuring a network infrastructure with a self-organizing concept [12]. The research suggests a self-organizing reconfiguration method of opportunistic infrastructure-mode in ad-hoc WiFi networks. In this network, each device can be either an access point or a client, and can change its role and wireless channel over time. 

As we have seen, these self-organizing concepts are fairly appropriate to a distributed and multi-agent system. In addition, ant colony optimization (ACO) algorithm [13] was adapted from a self-organization concept in biologic systems. ACO was inspired by the real-life behavior of a colony of ants when they go looking for food. Each ant releases a pheromone onto its path way while looking for food and the pheromones on the shorter paths soon increases. Therefore, ambulatory ants are capable of finding the shortest path between a food source and the nest by determining the amount of pheromone on the path. The pheromone trail from the ACO algorithm can be applied to various application fields such as network routing [14], routing of vehicles [15], robot navigation [16], and so on, with dynamic path environments. In particular, there is research about service discovery in pervasive environments [17,18]; but that research based on service queries through network hops is not based on real devices.

## 4. SoSp Architecture

### 4.1. Configuring the SoSp Network Environment 

**Figure 4:**
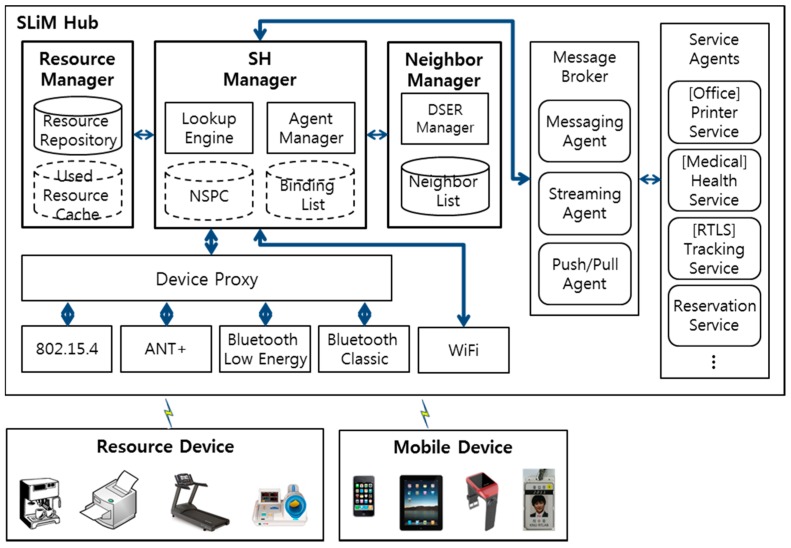
SoSp composition and SLiM Hub software architecture.

The proposed SoSp network architecture comprises SLiM Hubs that represent unit spaces, resource devices used to provide physical services, and the mobile devices with which users request services. Figure 4 shows the proposed software architecture of the SLiM Hub and the composition of the SoSp.

Resource devices such as office equipment, home appliances, and health equipment have the capability to link with an iLBS through wired or wireless communications with SLiM Hubs which are installed with communication modules in the unit spaces. A user can easily request any iLBS from the physical resources (such as printing, health checks, and hot coffee) with a mobile device (such as a watch, smartphone, or smart pad) using its wireless communication function (e.g., WiFi, Bluetooth, *etc.*). The mobile devices and resource devices always communicate with the SLiM Hub in the unit space using localization protocol, which provide real-time localization and the ability to transfer asynchronous messages amongst numerous mobile nodes, such as mobile devices and service resource devices.

The proposed SoSp consists of the SLiM Hub, resource devices, and mobile devices in an overlay network platform of two-tier architecture (*i.e.*, a stationary node and mobile nodes). This concept of the SoSp has been adapted for a self-organizing and fully distributed network infrastructure providing scalability and availability, unlike a centralized server-based architecture.

### 4.2. Previous Research: Protocols for Proposed Services 

**Figure 5:**
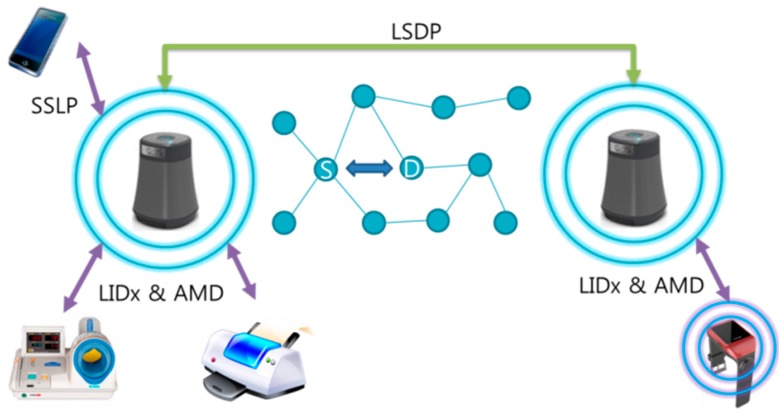
Protocols to support iLBSs in the proposed platform.

In order to support the proposed services, specific designed protocols for localization of the mobile devices and between numerous devices and SLiM Hubs are needed. As shown in Figure 5, the proposed self-organizing distributed platform works using three protocols that were developed with our previous research: location-ID exchange & asynchronous message delivery (LIDx & AMD), location-based service discovery protocol (LSDP), smart device to SLiM Hub service lookup protocol (SSLP).

There are many indoor location systems using various wireless indoor positioning solutions [19]. In particular, the LIDx location protocol is based on a cell-based location positioning technique. The LIDx & AMD [20,21] protocol can provide real-time localization for numerous mobile and resource devices in a complex and dynamic indoor environment, such as a hospital, warehouse, or museum. This localization protocol uses a simple bidirectional communication channel between the SLiM Hub and mobile devices, so it guarantees efficient movement of mobile devices despite having to support real-time tracking of numerous mobile devices. Each SLiM Hub in the unit space periodically broadcasts a beacon message with an ID to all mobile devices within its range. Each mobile device receives and compares the received signal strength indication (RSSI) value from each SLiM Hub, then exchanges IDs with the SLiM Hub that sends a better value (meaning the SLiM Hub is closer to the mobile device). The LSDP [22] for discovery of a needed resource or service supports an iLBS. The SLiM Hub uses only information about its connected resource within its range and neighboring SLiM Hub, and can look for the requested resource or service using the LSDP. This cell-based SLiM Hub determines the adaptive services and resources using only the information regarding its neighbors through cooperation amongst neighboring SLiM Hubs. In the proposed iLBS platform, a smartphone or smart pad can connect with the SLiM Hub using the SSLP [23], and request or receive needed iLBSs using the WiFi or Bluetooth protocols.

### 4.3. Software Architecture of the SLiM Hub 

The internal software architecture of the SLiM Hub, which is the service agent and router for the distributed environment, comprises five components: the Device Proxy, the Resource Manager, the Neighbor Manager, the SLiM Hub (SH) Manager, and the Service Agents.

The Device Proxy analyzes communication data through the wireless communication module, after which it forwards the analyzed data to the requested Service Agent. This Device Proxy offers multifarious wireless communication protocols, including 802.15.4, Bluetooth, and ANT+, but not WiFi. Mobile devices using WiFi have the capability to communicate directly with the SH Manager; however, other mobile devices are capable of communicating through the Device Proxy.

The Resource Manager manages and stores in a Resource Repository the usable services and resources of the Service Agent running in the SLiM Hub. The numerous mobile and resource devices obtain information from the Device Proxy using the LIDx & AMD [20] protocol, which are also stored and managed in the Resource Repository. In addition, resources used more than once are stored in a Used Resource Cache (URC). The used resources are stored and counted when these are reused in the URC. If the stored resource in the URC is not used anymore, the resource is no longer managed. Hence, the proposed architecture is highly efficient as time passes due to the fact that its lookup history is cached. The Resource Manager also manages the default configuration and the core resources for the SLiM Hub. The SH Manager handles inquiries for the requested service or resource with the help of the Resource Manager.

The Neighbor List consists of the physical neighbors connected through a geographic path, such as a door or a corridor, or logical neighbors grouped by a user’s living spaces, such as a house or an office. The distance values between the physical neighbors are calculated and determined by the mobile device traveling between the SLiM Hubs through the geographic path. The Neighbor Manager connects neighboring SLiM Hubs in the above manner and adds information about neighboring SLiM Hubs to the Neighbor List. In addition, the Dynamic Space Expanding and Reducing (DSER) Manager periodically sends a heartbeat message to all SLiM Hubs in the Neighbor List for detecting a removed neighboring SLiM Hub from current infrastructure, and provisionally has its neighbors’ Neighbor List for responding. The detailed installation or deletion processes using heartbeats and neighbor information are detailed in Section 5.3.

The SH Manager, working as the Agent Manager, controls the lifecycle of all Service Agents capable of running in the SLiM Hub. The mobile devices or resource devices with WiFi protocol directly interact with this SH Manager using the Ice [24] interface for a distributed platform. In addition, the SH Manager provides lookup results based on the proposed Lookup Engine, details of which are discussed in Section 5. The Lookup Engine also maintains and manages connections with the mobile and resource devices in order to support stable and continuous services using the Binding List, which includes binding information.

The Service Agents, which are related to the service resources and service applications for the SLiM Hub, manage the requested service data and communicate with resource devices through the wireless communication module in the unit space. There are core Service Agents managed by the Resource Manager, and core services can always be provided in the current SLiM Hub. These core services have particular characteristics depending on each individual SLiM Hub installed in the various locations.

In order to provide various services via Service Agents, the SLiM Hub needs the core functions to support the requested services. For example, in a medical service system, patients or staff members can request a health data anywhere and anytime. In order to send the requested health data to the user, a messaging function is always required in the SLiM Hub middleware, or a streaming function is required for continuously receiving the health data from wearable medical equipment. Also, in order to send and receive the data anywhere and anytime, the push/pull function is needed. These core functions must always be supported.

## 5. Self-Organizing Dynamic Space Configuration

### 5.1. Configuring the SoSp Overlay Network 

**Figure 6:**
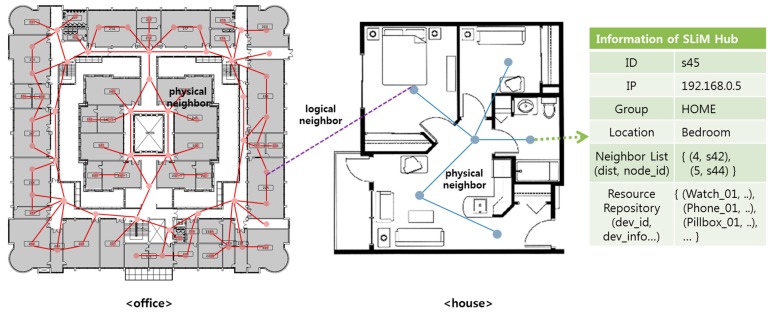
Physical and logical neighbors.

All SLiM Hubs are connected to other SLiM Hubs through a network, such as Ethernet, that does not reflect geographic location. In order to reflect these physical paths, the proposed opportunistic iLBS based on an overlay network with the shortest geographic distance between neighbors discovers the required resources using information about these neighboring SLiM Hubs in a self-organizing and distributed environment. Figure 6 illustrates examples of physical and logical neighbors. The SLiM Hub is connected to physical neighbors associated with the geographic path, or logical neighbors grouped by user. Each SLiM Hub only knows the distance and information of physical neighboring SLiM Hubs. In addition, the SLiM Hub manages mobile or stationary resource devices such as watches, phones, or pill-boxes within range of the unit space.

A physical neighbor is connected through a geographic path, such as a door or a corridor. To search for the geographically nearest resource to the current location, the Lookup Engine discovers the requested resource based on the shortest path according to the distance between the physical neighbors [6]. The distance value between the physical neighboring SLiM Hubs is calculated and determined by the mobile device traveling between the SLiM Hubs through the physical path [25]. In order to connect with the physical neighboring SLiM Hub, the mobile device can use three methods: (1) a mobile user manually configures the mobile device; (2) by the movement of the mobile device; or (3) either manual or automatic connection is established.

The first method is when a mobile device user manually and directly connects with two SLiM Hubs on the physical path via the device’s display. All information for each SLiM Hub is already saved in the user’s mobile device.

**Figure 7:**
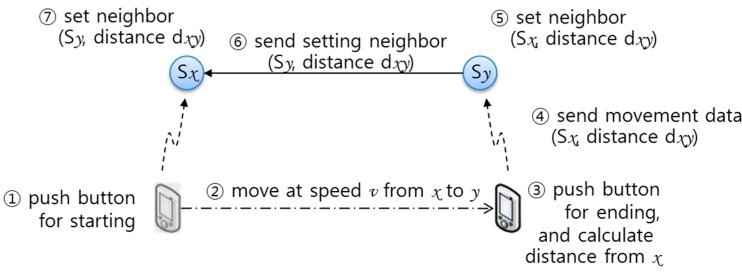
Manual method for connecting physical neighboring SLiM Hubs.

Using the second method, the mobile user manually pushes a button to configure neighbors at the first and next SLiM Hub points, receiving the information from the current SLiM Hub, as shown in Figure 7. In this process, the mobile user needs to walk at a steady pace. In this scenario, we assume that a person walks at speed of 1.2 m/s, and we estimate distance using this constant value. A SLiM Hub receives the connection message for another SLiM Hub from a mobile device and sends the connection message through itself to the connected neighboring SLiM Hub.

**Figure 8:**
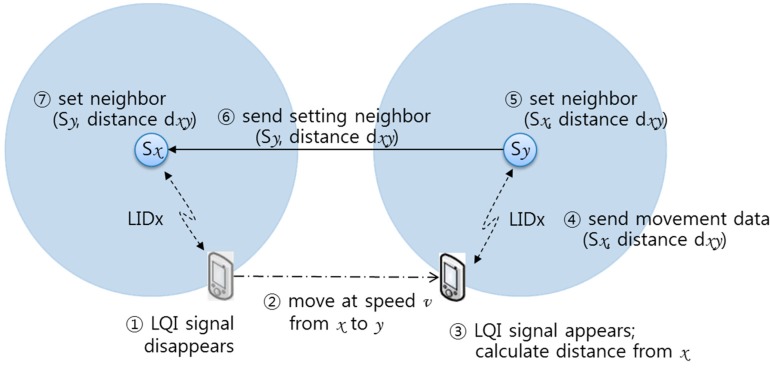
Automatic method to connect physical neighboring SLiM Hubs.

The third method, which also accommodates movement of a mobile device, is an automatic connection method via LIDx protocol between a mobile device and SLiM Hub. Fundamentally, the mobile device communicates with the SLiM Hub using the LIDx protocol for real-time localization [20]. The SLiM Hub with mains power periodically broadcasts a beacon message. The mobile device can check a link quality indicator (LQI) signal from the SLiM Hub. Although the LQI fluctuates from various forms of interference, it has a particularly strong value within 3 m of a SLiM Hub [25]. Thus, the mobile device can use the LQI signal to estimate its distance from a SLiM Hub. Therefore, the mobile device can calculate distance using the LQI signal from the SLiM Hub and the walking speed used in the manual method, as shown in Figure 8.

In this automatic process, a mobile device calculates the distance from the previous SLiM Hub using Formula (1), where $d_{xy}$ is the value of the distance pertaining to moving between SLiM Hub *x* and SLiM Hub *y* when a mobile user walks at steady speed of $v_{walk}$ during $T_{walk}$ time. The parameters $d_{LQI_{x}}$ and $d_{LQI_{y}}$ are estimated using the LQI signal from each SLiM Hub within range of the SLiM Hub’s signal. If the LQI signal from the current SLiM Hub disappears, the mobile device checks and starts to calculate time pertaining to walking until the signal reappears from the other SLiM Hub.

(1) $$d_{xy}=d_{LQI\_x}+d_{LQI\_y}+(\upsilon_{walk}*T_{walk})$$

On the other hand, a logical neighbor is not directly connected through a geographic path and is a geographically distant SLiM Hub grouped by the user’s living space, such as a house or a hospital. This logical neighbor SLiM Hub, which is authorized and registered by a smartphone user, can be useful in providing the individual with resources related to the user’s life pattern.

### 5.2. Adaptable Overlay Network in Dynamic Environment

**Figure 9:**
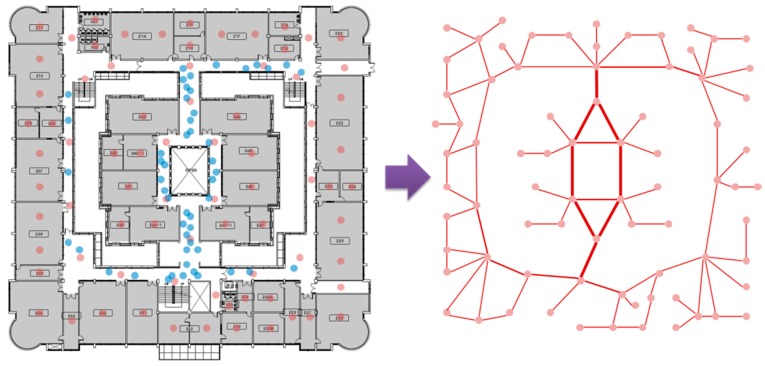
Dynamic map based on the movement of mobile users in the proposed overlay network.

The method of configuring a physical path by mobile devices can be used to support an adaptable service in a dynamic environment. A building environment such as a hospital is highly dynamic, with numerous passers-by in the indoor surroundings. In particular, this factor can be applied to provide an adaptable iLBS depending on the type of service. For instance, when the existing path is temporarily blocked, a path for supporting an iLBS must dynamically change. Furthermore, it may need the path for additional or fewer people depending on the type of service. Figure 9 shows the dynamic path (red lines) and map based on the movement of mobile users (blue circles) in the proposed overlay network.

A dynamic path is determined using an ACO algorithm [13] inspired from the behavior of ants in the natural world. In the proposed platform, mobile users (like ants) deposit a virtual “pheromone” on the paths recorded by the localization algorithm using the LIDx protocol [20]. The pheromone trail has two factors: increments by more users and evaporation from the flow of time. Therefore, the amount of pheromone in the proposed platform is updated with Formula (2), where the amount of pheromone $\tau_{xy}$ is a weight of the path between SLiM Hub *x* and SLiM Hub *y*, ρ is the pheromone evaporation coefficient from the flow of time, and $\tau_{xy}^{k}$ is the amount of pheromone deposited by the k^th^ mobile user. All increments of pheromone from each mobile user have the same value: 1.

(2) $$\tau_{xy}\leftarrow (1-\rho )\tau_{xy}+\sum \tau_{xy}^{k}$$

**Figure 10:**
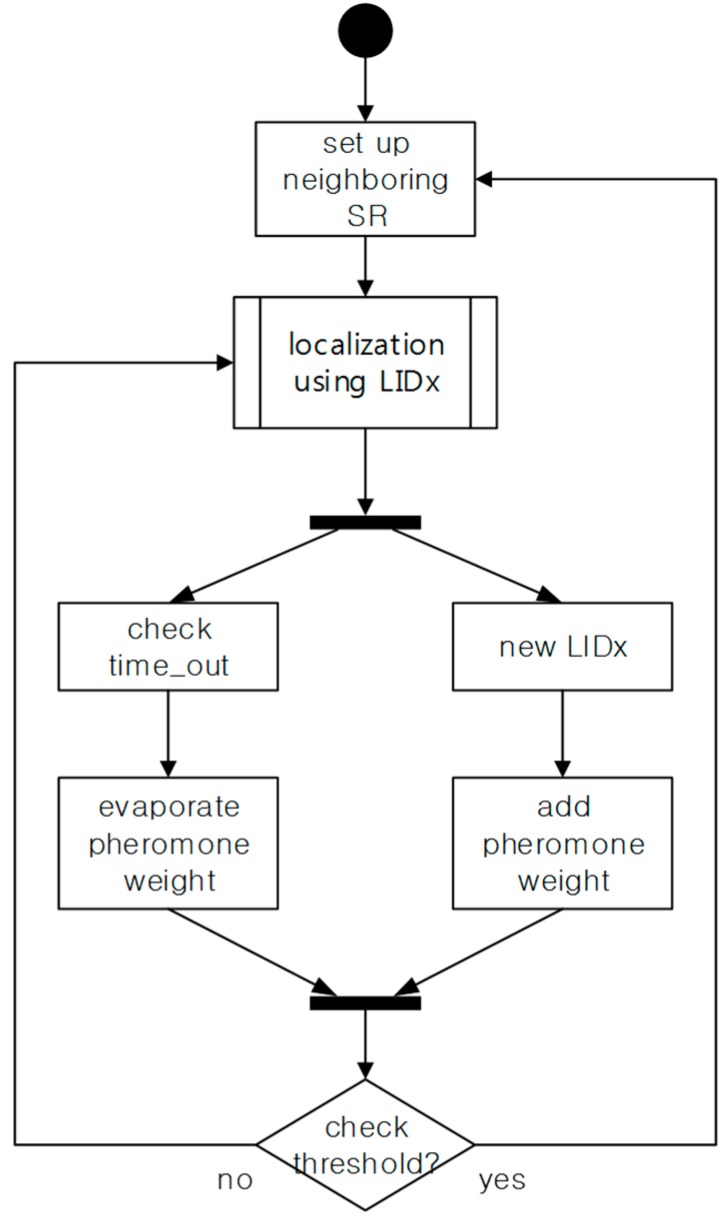
The state diagram from the update process for the pheromone trail.

Figure 10 shows the state diagram of the update process from the above factors. Through the localization process using LIDx, the weight of the path increases or decreases. In particular, the value of neighboring information needs updating because the paths of users are extremely dynamic. Therefore, if the updated pheromone exceeds the threshold value, the pheromone weight is reflected in the distance value of the path.

### 5.3. Dynamic Space Expansion and Reduction

The proposed self-organizing platform supports scalability of the infrastructure in a dynamic environment. In order to reconfigure the changed infrastructure without global knowledge, the Neighbor List and neighbor shortest path cache (NSPC) based on geographic distance paths in each SLiM Hub should be adaptable and flexible to dynamic surroundings such as insertion of a new SLiM Hub, or deletion or re-activation of an existing SLiM Hub.

#### 5.3.1. Heartbeat Based Neighbor Periodic Status Monitoring

**Figure 11:**
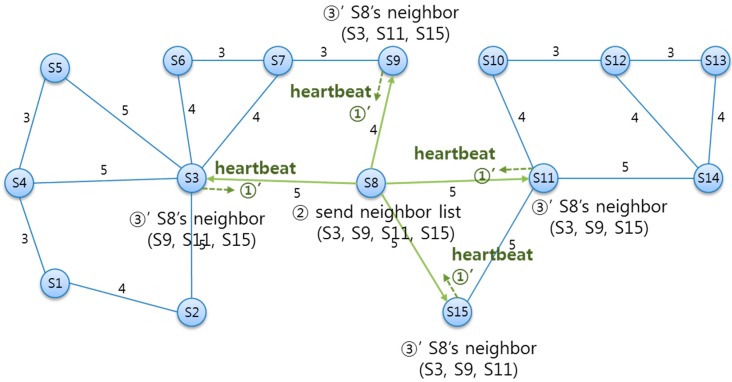
The periodic heartbeat process to check for a deleted SLiM Hub.

The event of deleting an existing SLiM Hub can occur at any time, under both normal and abnormal situations. Therefore, a SLiM Hub periodically monitors the status of all neighboring SLiM Hubs using a heartbeat message. Figure 11 shows the process of sending the heartbeat message, and receiving the neighbors’ Neighbor List. This information of two-hop neighbors is used for connecting to a physical path that excludes the deleted SLiM Hub. The monitoring procedure via heartbeat message is the following process. ① Each SLiM Hub periodically sends a heartbeat message to all neighboring SLiM Hubs. ② The neighbor SLiM Hubs that receive the heartbeat message send the current Neighbor List in response. ③ Each SLiM Hub that receives the neighbors’ neighbor lists provisionally retains this information. In cases of normal removal from the current infrastructure, a deleted SLiM Hub can also send a removal message and the current Neighbor List without receiving a heartbeat message. However, a deletion is almost always an abnormal situation, such as a sudden shutdown, which can occur at any time and at any position. In addition, redirected paths to other existent physical SLiM Hubs are needed using neighbor information of the deleted SLiM Hub.

#### 5.3.2. Inserting New SLiM Hub

**Figure 12:**
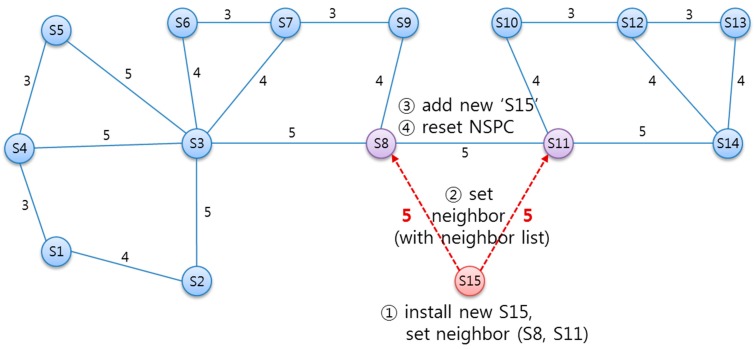
The state diagram for installing a new SLiM Hub.

**Figure 13:**
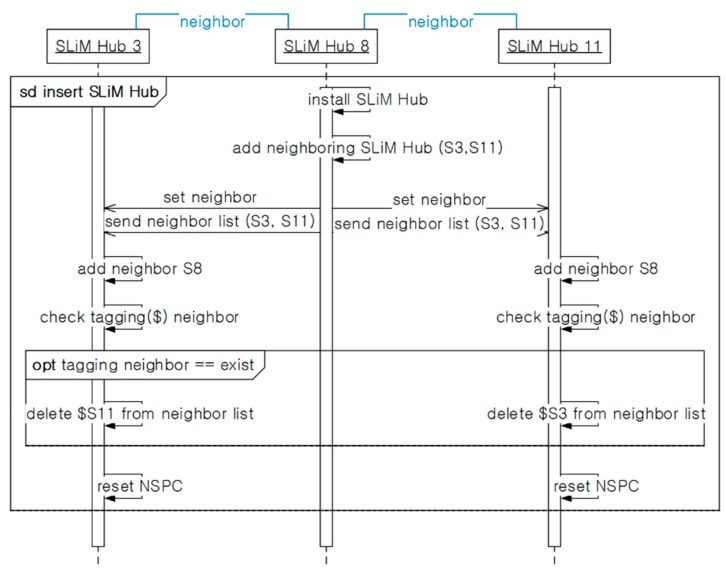
The sequence for installing or reinstalling a SLiM Hub.

Figure 12 shows the process of installing a new SLiM Hub. ① If new SLiM Hub ‘S15’ is inserted with physical paths into the existing infrastructure, SLiM Hub ‘S15’ can add to its Neighbor List to both ‘S8’ and ‘S11’ either automatically or manually. ② The inserted ‘S15’ requests connection to physically neighboring SLiM Hubs ‘S8’ and ‘S11’ and sends its own neighbor information. If the inserted SLiM Hub has been reinstalled after a temporary deletion, the neighbors’ information is used for comparison with redirection paths. (The detailed procedure for reinstalling is described with the next scenario for deletion of an existing SLiM Hub.) ③ The SLiM Hubs that receive the message from new SLiM Hub ‘S15’ adds the information about ‘S15’ to its current Neighbor List. ④ Finally, the current NSPC in each SLiM Hub is reset. Figure 13 shows the process for installation and reinstallation. This sequence is run for the reinstallation process after a temporary deletion.

#### 5.3.3. Deleting an Existing SLiM Hub

**Figure 14:**
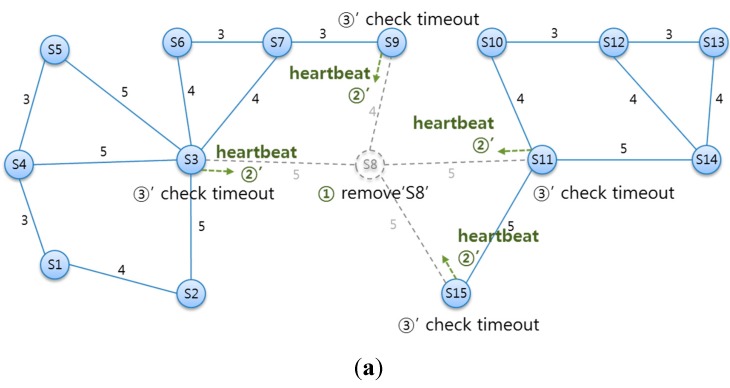

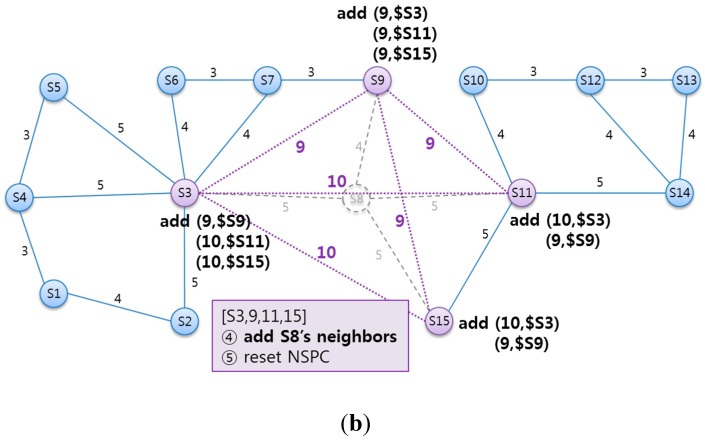
(**a**) Checking for a deleted SLiM Hub by heartbeat message; (**b**) Creating new paths to existent physical SLiM Hubs, notwithstanding of deleted SLiM Hubs.

With the above heartbeat process, SLiM Hubs can monitor the status of neighboring SLiM Hubs. Figure 14 illustrates checking for deleted SLiM Hubs via heartbeat message and creating new paths to existing SLiM Hubs. As shown in Figure 14, ① if SLiM Hub ‘S8’ is temporarily or arbitrarily removed from the current infrastructure, ② no neighboring SLiM Hubs to ‘S8’ will receive a response to a heartbeat message. ③ Each SLiM Hub starts the timeout count with the heartbeat request, and checks for the deleted neighboring SLiM Hub ‘S8’. The SLiM Hubs checking for the deleted SLiM Hub need to create new paths to existent physical SLiM Hubs, notwithstanding the deleted SLiM Hub. ④ Therefore, each SLiM Hub uses the information of its neighbors’ neighbor lists, and connects to these SLiM Hubs as new neighbors. In this process, the new neighbors are temporarily tagged as redirected neighboring SLiM Hubs. These tagged SLiM Hubs are compared with the neighbor lists of a reactivated SLiM Hub later. ⑤ Finally, the current NSPC in each SLiM Hub is reset.

**Figure 15:**
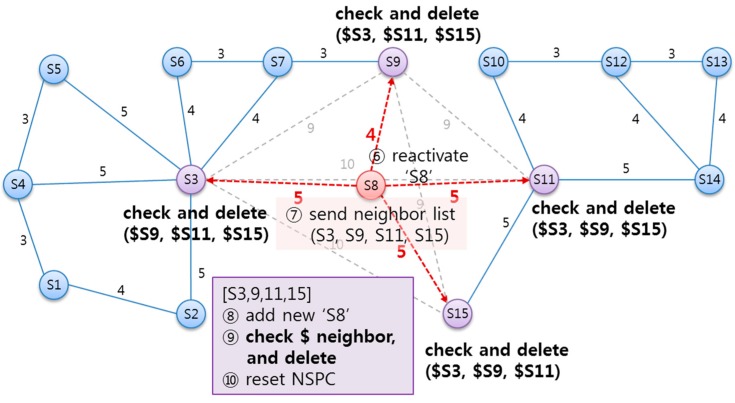
The state diagram from reactivation of an existing SLiM Hub after transient deletion.

As illustrated in Figure 15, if a SLiM Hub is temporarily removed from the infrastructure, ⑥ this SLiM Hub can be reactivated in its original position. The reactivation procedure is the same as the installation procedure above. ⑦ The reactivated SLiM Hub ‘S8’ requests connection to the physical neighboring SLiM Hubs and sends its own neighbor information. ⑧ The SLiM Hub that receive the reactivation message from SLiM Hub ‘S8’ add the information about ‘S8’ to the current Neighbor List. ⑨ This SLiM Hub checks the distance and path of redirected neighbors with the inserted SLiM Hub’s neighbors. If the created new path from the inserted SLiM Hub already exists in a redirect path, this redirect path is deleted from the current Neighbor List. ⑩ Finally, the current NSPC in each SLiM Hub is reset.

**Figure 16:**
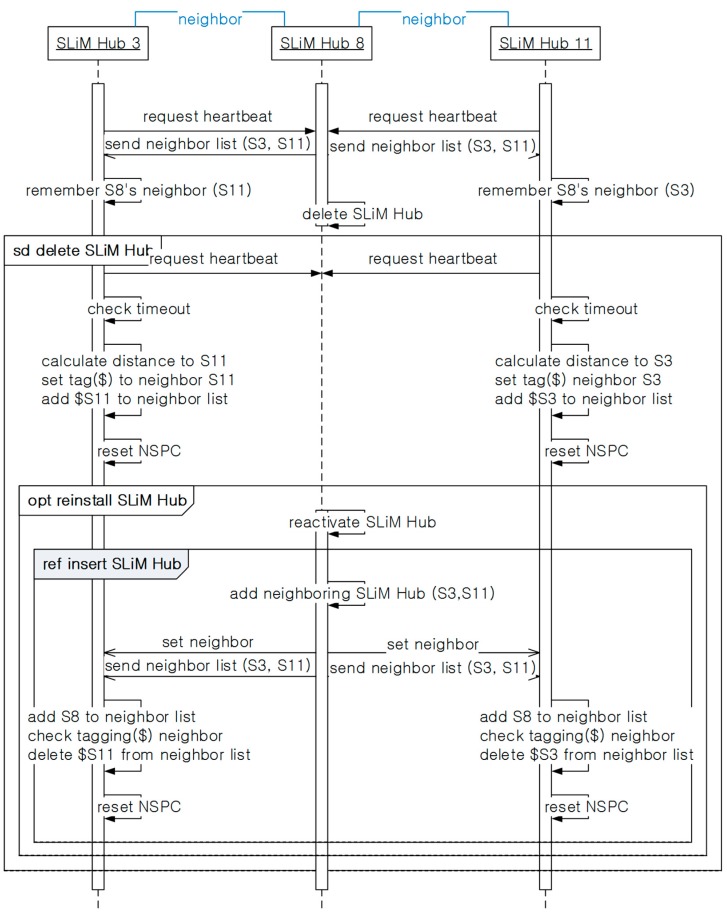
The sequence of installing or reinstalling a SLiM Hub.

Figure 16 shows the overall sequence diagram, including the above processes for deletion and reactivation of an existing SLiM Hub. All SLiM Hubs periodically send a heartbeat message, and receive the neighbor lists in response. Via the heartbeat process, each SLiM Hub can detect deleted neighboring SLiM Hubs. Then, SLiM Hubs checking for the deleted neighbor add the existent physical SLiM Hub tagged with a redirect path to the current Neighbor List. If the deleted SLiM Hub is reactivated at the original position, the reactivated SLiM Hub runs the insertion process seen in Figure 13. In case of reactivation other than in the installing process, restoration of the old path is needed. Therefore, each SLiM Hub checks and deletes the tagged redirect paths from the current neighbor lists.

**Figure 17:**
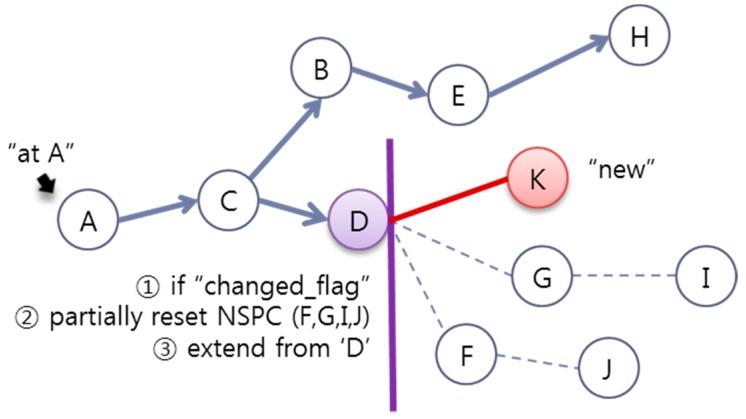
The procedure for changing the NSPC when there are changes at two-hop or more SLiM Hubs.

The one-hop neighboring SLiM Hubs from the changed SLiM Hubs can easily be detected via the insertion message or the heartbeat message, and can reset the current NSPC. However, a two-hop or more SLiM Hub cannot always monitor the status of all SLiM Hubs in the NSPC. In addition, this is unnecessary because they are used in the lookup process. Therefore, a SLiM Hub running the lookup process checks for the ‘changed_flag’ at the particular SLiM Hub in the current NSPC list, and partially resets and extends the current NSPC, as shown in Figure 17.

## 6. Implementation and Evaluation

### 6.1. SLiM Hub and Mobile Device Hardware/Software Implementation

**Figure 18:**
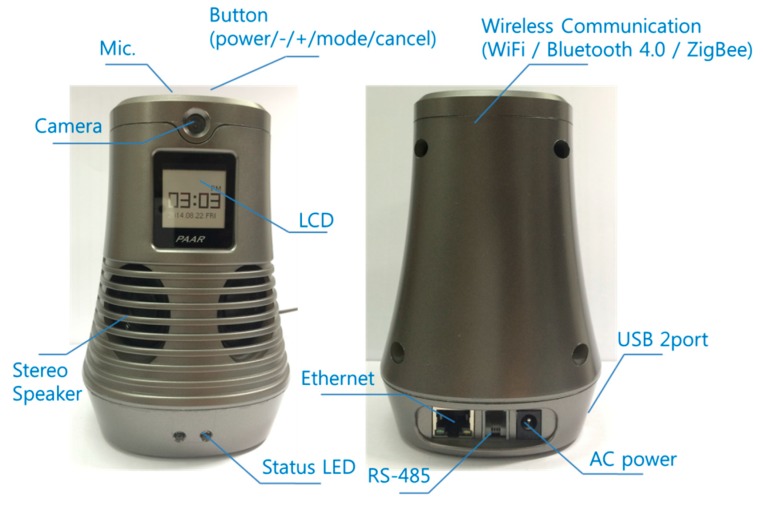
The SLiM Hub hardware with its various wireless and wired communication modules.

SLiM Hubs are installed in every unit space of a building, such as a room or a corridor with a wired communication protocol such as Ethernet and various wireless communication protocols such as WiFi, ZigBee, Bluetooth 4.0 (dual mode), and ANT+. The SLiM Hub hardware, as shown in Figure 18, is embedded various wireless communication modules. The SLiM Hub utilizes a wired 10/100 Ethernet and RS-485 communication port for the constituent infrastructure of the iLBS system as well as two USB 2.0 ports for USB storage, USB resource device (*i.e.*, USB printer) or other wireless communication modules in addition to Bluetooth 4.0, ZigBee and WiFi.

In order to attain the cooperation needed between the neighboring SLiM Hubs and mobile devices using the wired/wireless networks in a self-organizing and fully distributed environment, internal software and smartphone application components are implemented using Ice [24], a distributed platform middleware. Ice facilitates the development of heterogeneous distributed applications by supporting various languages, such as C++, Java, C#, Python, Object-C, Ruby, and PHP over Windows, Linux, iOS, and Android platforms.

**Figure 19:**
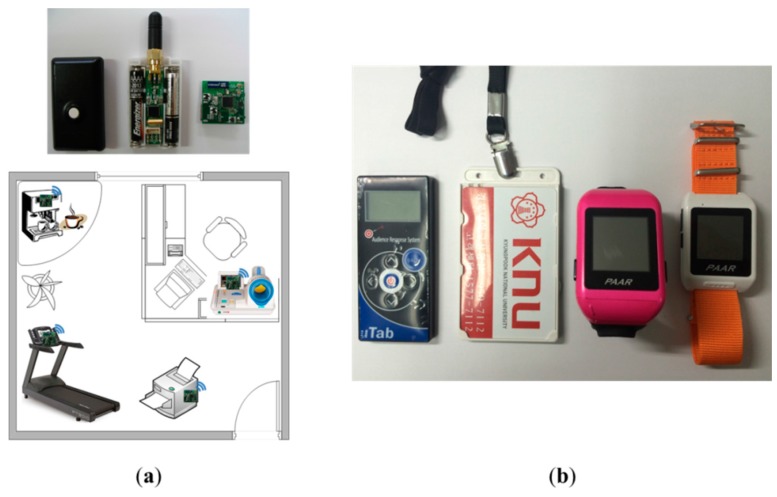
Service resource devices and mobile devices owned by users. (**a**) Service resource devices with communication module; (**b**) Mobile devices.

There are various types of cell-based SLiM Hubs and mobile devices using wireless communications in the infrastructure. Resource devices, such as office equipment, home appliances, and health equipment in the unit space can provide real-time localization using the LIDx & AMD [20] wireless communication protocol developed by our research team. Mobile devices use diverse types of smart watches, smartphones, or smart tags. Mobile devices provide an adaptive service using an appropriate profile for attributes of the devices through the connection with resource devices. In particular, ID matching with a mobile device owned by a user is important for resources requiring privacy, such as medical and health equipment; users enable a request to a service using their individual mobile devices. Figure 19 shows the communication modules built into the resource devices and various types of mobile devices owned by users.

### 6.2. Dynamic Space Test and Evaluation through Simulation

The proposed architecture supports a dynamic space expanded and reduced via the DSER manager in a dynamic environment. In order to evaluate insertion and deletion using DSER management, an infrastructure for such scalability was configured using dynamic reconfiguration of a log of SLiM Hub processes. The SLiM Hub processes were dynamically inserted into or deleted from an existing infrastructure, and neighboring SLiM Hubs were reconfigured using a method of expansion and reduction to support scalability with a self-organization property.

**Figure 20:**
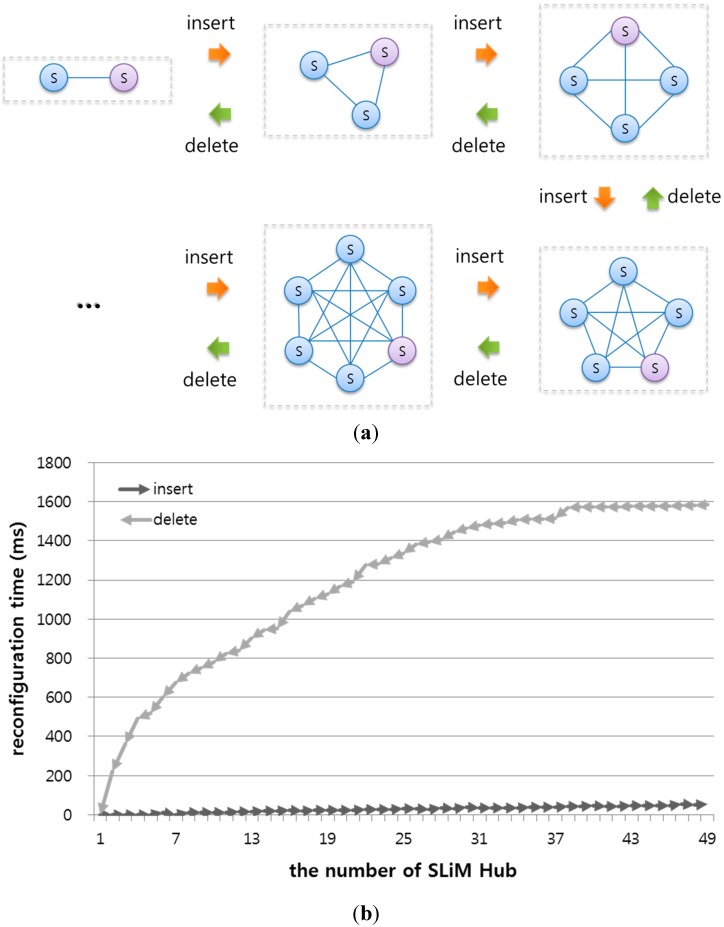
Reconfiguration evaluation from inserting or deleting SLiM Hubs. (**a**) Process of inserting or deleting one SLiM Hub for fully connectivity. (**b**) Reconfiguration times when inserting or deleting up to 50 SLiM Hubs.

Figure 20 shows the insertion and deletion procedures for the evaluation and reconfiguring times of an infrastructure during the insertion or deletion processes. First, an infrastructure with one SLiM Hub was expanded to 50 SLiM Hubs. Then, an infrastructure with a total of 50 SLiM Hubs was reduced to one SLiM Hub. Each inserted or deleted SLiM Hub had full connectivity with all existing SLiM Hubs. The insertion time refers to the time to insert each SLiM Hub. However, the deletion process has heartbeat time for all connected neighboring SLiM Hubs.

**Figure 21:**
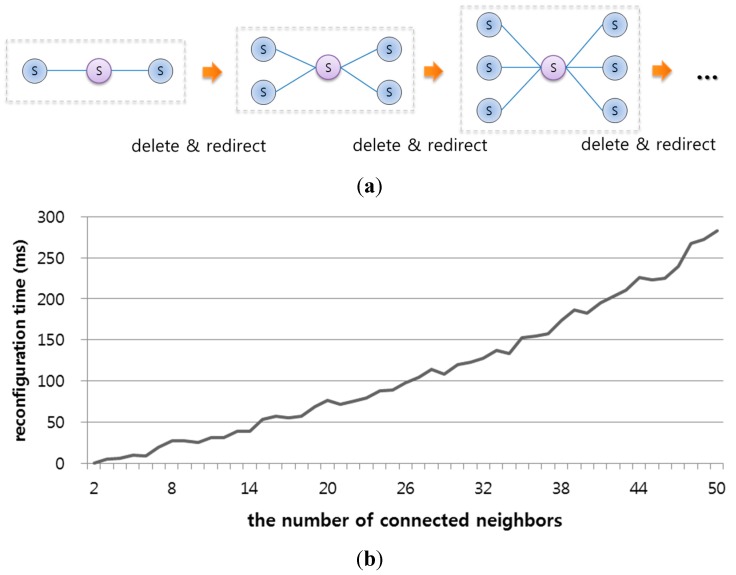
Reconfiguration evaluation for deletion and reactivation of one SLiM Hub with between one and 50 neighbors. (**a**) Infrastructures for an evaluation of deletion and reactivation; (**b**) Reconfiguration time for deletion and reactivation.

Figure 21 shows the reconfiguration time for an infrastructure during the deletion and reactivation processes. The infrastructure with one point of contact was configured with from two to 51 SLiM Hubs. One SLiM Hub in contact with the others had the number of from two to 50 connected neighbors. When one SLiM Hub was deleted, the connections between the other SLiM Hubs were created. The reconfiguration time for deletion and reactivation of one SLiM Hub was less than the evaluation from insertion and deletion via heartbeat messages between all connected SLiM Hubs because an infrastructure with one point of contact has fewer neighbors and connections than a fully connected infrastructure.

## 7. Conclusions

An infrastructure for an iLBS in a dynamic indoor environment is necessary for an efficient architecture that reflects a different approach to a traditional centralized platform. This paper suggests a method for dynamic space expansion and reduction based on the proposed self-organizing and fully distributed platform. The nodes in the iLBS can determine insertion or deletion at any time, and the infrastructure must be reconfigured through the self-organizing concept without influencing the overall infrastructure under transient circumstances. In addition, the proposed self-organizing platform supports scalability of physical space or a logical service group. We evaluated the reconfiguration time of the infrastructure during dynamic insertion or deletion processes in the proposed self-organizing distributed platform, and showed that the proposed platform is efficient in providing the dynamic space expansion and reduction.

The proposed SoSp with SLiM Hub platform enhances scalability, decentralization, fairness, and robustness whenever numerous mobile devices concurrently request resource lookups from several locations. In addition, there is no need to maintain a map of the entire indoor location, unlike traditional centralized server methods. Future research will focus on extending advanced iLBS, such as an indoor navigation system.
